# Eight Children at a Birth

**Published:** 1872-11

**Authors:** 


Eight Children at a birth.—On the 24th of Aug., Mrs. Timothy Bradlee, of Trumbull Co., Ohio, gave birth to eight children—three girls and five boys. They are all living, and are healthy, but quite small. Mr. Bradlee was married six years ago to Eunice Mowery, who weighed 273 pounds on the day of her marriage. She has given birth to two pairs of twins, and now eight more, making twelve children in eight years. Mrs. Bradlee was a triplet, her mother and father being twins, and her grandmother the mother of five pairs of twins.—*Ex.*

This story, which has been going the rounds of most of our exchanges, started several years ago as a joke; since its revival this time it has been denied by parties acquainted with the history of the case.—*Ed.*

